# Decision regret among couples experiencing infertility: a mixed methods longitudinal cohort study

**DOI:** 10.1186/s12978-023-01699-5

**Published:** 2023-11-09

**Authors:** Rachel Cusatis, Colin Johnson, Katherine D. Schoyer, Shirng-Wern Tsaih, Joanna Balza, Jay Sandlow, Kathryn E. Flynn

**Affiliations:** 1 Department of Medicine, Medical College of Wisconsin, 9200 W. Wisconsin Ave, Milwaukee, WI USA; 2 Department of Obstetrics and Gynecology, University of Iowa Hospitals and Clinics, Iowa City, IA 52242 USA; 3 Department of Obstetrics and Gynecology, Medical College of Wisconsin, Milwaukee, USA; 4 Institute for Health and Equity, Medical College of Wisconsin, Milwaukee, USA; 5 Department of Urology, Medical College of Wisconsin, Milwaukee, USA

**Keywords:** Decision-making, Decision regret, Decisional regret scale, Infertility, Fertility treatment, Mixed methods

## Abstract

**Background:**

Decisions for how to resolve infertility are complex and may lead to regret. We examined whether couples and individuals who sought a consultation from a reproductive specialist for infertility later expressed decisional regret about their family-building choices and whether regret was associated with parental role, family-building paths, or outcomes.

**Methods:**

This longitudinal mixed methods study included women and their partners who completed a questionnaire prior to their initial consultation with a reproductive specialist and 6 years later. The six-year questionnaire included the Ottawa Decision Regret Scale referencing “the decisions you made about how to add a child to your family.” A score of 25+ indicates moderate-to-severe regret. Additional items invited reflections on family-building decisions, treatments, and costs. A systematic content analysis assessed qualitative themes.

**Results:**

Forty-five couples and 34 individuals participated in the six-year questionnaire (76% retention rate), Half (n = 61) of participants expressed no regret, which was similar by role (median 0 for women and supporting partners, F = .08; p = .77). One in 5 women and 1 in 7 partners expressed moderate-to-severe regret. Women who did not pursue any treatment had significantly higher regret (median 15; F = 5.6, p < 0.01) compared to those who pursued IVF (median 0) or other treatments (median 0). Women who did not add a child to their family had significantly higher regret (median 35; F = 10.1, p < 0.001) than those who added a child through treatment (median 0), through fostering/adoption (median 0), or naturally (median 5). Among partners, regret scores were not associated with family-building paths or outcomes. More than one-quarter of participants wished they had spent less money trying to add a child to their family. Qualitative themes included gratitude for parenthood despite the burdensome process of family-building as well as dissatisfaction or regret about the process. Results should be confirmed in other settings to increase generalizability.

**Conclusion:**

This longitudinal study provides new insight into the burden of infertility. For women seeking parenthood, any of the multiple paths to parenthood may prevent future decision regret. Greater psychosocial, financial, and decision support is needed to help patients and their partners navigate family-building with minimal regret.

**Supplementary Information:**

The online version contains supplementary material available at 10.1186/s12978-023-01699-5.

## Plain Language Summary

When people experience infertility, there are many decisions that can be challenging, such as whether to seek fertility treatments, to pursue fostering/adoption, and how to manage costs. With each decision, there is an opportunity for regret. The goal of this study was to look at whether people who were experiencing infertility and made an appointment with a doctor who specializes in infertility felt any regret about their decisions 6 years later. We also looked at whether different roles (that is, women seeking pregnancy or their supporting partners), different family-building paths (that is, medical treatments or not), or different outcomes (that is, adding a child to their family or not) were associated with different levels of regret. Results showed that half of the 120 people in the study did not have any regret 6 years after meeting with a specialty doctor. However, some patients did have regret, including 20% of women and 14% of partners who expressed moderate-to-severe regret. Women who did not add a child to their family in the six years during the study reported higher regret compared to women who did add a child to their family. There were no such differences among partners. About 25% of participants wished they had tried more, fewer, or different treatments. More than 25% wished they spent less money to try to add a child to their family. For people who want to add a child to their family, there are multiple ways to become a parent, any of which may be linked to lower decision regret. Decision regret is experienced differently between women seeking to add a child to their family and their partners. Would-be parents need more emotional, financial, and decision making support to help them navigate family-building with minimal regret.

**Supplementary Information:**

The online version contains supplementary material available at 10.1186/s12978-023-01699-5.

## Background

The impact of infertility, or the inability to become pregnant after 12 months of unprotected intercourse [1] extends beyond sexual and reproductive facets to include psychosocial and quality of life implications [2–5]. People experiencing infertility have exhibited stress and distress, [3, 6, 7] decreased social well-being, [8, 9] and described impacts on personal and marital relationships [10]. Global estimates of infertility prevalence are similar by region and income level, ranging from 10 to 12% in Europe and the Americas to 13–16% in Asia and Africa [11]. However, many who experience infertility do not pursue treatment [12]; in the U.S., about 10% of women report having talked to a doctor about infertility, while only 4% have used ovulation medications and less than 2% have tried intrauterine insemination (IUI) [13]. Undergoing fertility treatment can be disruptive to many aspects of a patient’s life, including psychologically [5, 14, 15], socially [14], and with employment [16] as treatments and appointments assume priority. Emotional distress is a common reason for treatment discontinuation [17].

The burdens of an infertility diagnosis and of undergoing fertility treatments can be significant both for patients [5] and their partners [14]. For people in a relationship, decisions for how to resolve infertility require balancing both personal priorities as well as a partner’s priorities, including cost, chance of success, importance of a genetic connection, importance of experiencing pregnancy and childbirth, and treatment side effects [18]. Once people begin a particular path to parenthood, whether it’s continuing to try unassisted, using medical treatments, or pursuing fostering or adopting, there are additional decisions regarding how many times to attempt a particular path, whether to switch paths, or when to stop pursuing parenthood and pursue a child-free lifestyle [15, 19]. While some previous research suggests women drive treatment decision-making in infertility [20], other research supports significant partner contributions to medical help-seeking in infertility [21].

Much of the literature examining decision making in infertility is specific to in vitro fertilization (IVF), particularly decisions about discontinuation of IVF treatment [22–25] and disposition of cryopreserved embryos, i.e., using them for future attempts at pregnancy, donating them to another couple, donating them for research, or thawing and discarding them [26–29]. However, especially in the U.S., where insurance coverage for infertility treatments varies by state so is not guaranteed [30], IVF-specific decisions are relevant to a relatively small proportion of people. Therefore, broader research on infertility decision-making among those who do *and* do not receive care is needed.

Decision regret in the healthcare setting is a negative emotion involving distress or remorse after a healthcare decision [31]. Early theories on regret focused on regret from action or inaction, with some evidence that action is associated with more regret in the short term, and inaction is associated with more regret in the long term [32]. Connolly and Reb [33] developed a framework identifying sources of potential regret in health care: regret about an outcome, regret about the option(s), and regret about the process. Research on regret in infertility has found that women with infertility have more reproductive regrets than those without infertility [34]. Others have addressed regret among patients with cancer about cryopreservation, genetic testing of embryos, and fertility consultation, where results indicate fertility discussions before cancer treatment were associated with lower regret and higher patient satisfaction [35, 36]. Decision regret after IVF treatment failure has been associated with anxiety [37]. Understanding decision regret about family-building paths and how patients and their partners later evaluate their family-building decisions may help clinicians improve their counseling and ultimately minimize decision regret. Therefore, the objective of this study was to examine decision regret 6 years after an initial consultation with a reproductive specialist and whether parental role, path(s) pursued, or outcomes were associated with regret.

## Materials and methods

### Study design and setting

This was a prospective longitudinal mixed methods observational study conducted at a Midwestern US academic medical center. Previous manuscripts have been published using baseline data [14, 18, 19, 38]. This manuscript adds the 6-year longitudinal follow-up data.

### Participants and procedures

In 2013–2014, letters were mailed to 613 patients and their partners who had a first consultation with a reproductive specialist scheduled at least 1 week in the future and an address within 30 miles of the center. No follow-up attempts were made, given the short window in which to collect baseline data before the initial consultation. Additional eligibility screening of the 155 patients who responded to the invitation (response rate 25%) confirmed that patients had not previously tried IVF or had a child using assisted reproductive technology, leaving 111 eligible patients. Of these, 92 patients (82% acceptance rate) and 68 of their partners (total n = 160) enrolled and completed a self-administered questionnaire via Research Electronic Data Capture (REDCap) [39, 40] prior to their first consultation. Participants were compensated $25 for completion of the baseline questionnaire, which included sociodemographics, health, priorities for family-building, decision making, and fertility-related quality of life. In late 2019, the 156 original participants who had agreed to be contacted again were invited to complete a self-administered questionnaire that included repeated content from the baseline questionnaire plus family-building paths, family-building outcomes, reflections on family-building, and decision regret. Participants (n = 124, 78% retention rate) were compensated $50 for completion of the six-year questionnaire. The research was approved for scientific and ethical integrity by the Institutional Review Board of the Medical College of Wisconsin.

### Measures

The Decision Regret Scale [41] is a 5-item scale with Likert-type response options (strongly agree to strongly disagree) that are summed and scaled to 0–100, where 0 means no regret. Scores of 25 or more are considered to reflect moderate-to-severe regret [42]. Items ask about whether a decision was the right decision, whether one regrets the choices they made, whether they would make the same choice if given the chance, whether a choice did them harm, and whether they made a wise decision. When completing the Decision Regret Scale, participants were given the instruction, “Please think about the decisions you made about how to add a child to your family”. Participants were asked seven de novo yes/no statements regarding participants’ thoughts about the number and types of fertility treatments they received and about their financial investment in adding a child to their family (Additional file 1: Table S1). For each statement, patients were asked to “please describe” their answer using an open text box. The six-year questionnaire also included the open-ended prompt, “Please describe how you feel about the decisions you made to try to add a child to your family.”

Patient characteristics that were self-reported at baseline included parental role, education, age, race/ethnicity, general health, household income, relationship status, and decisional conflict. Decisional conflict can occur when competing options lead to uncertainty about a course of action to take, in the case of infertility these competing options relate to how to add a child to one’s family [42]. The decisional conflict scale is a 16-item measure that uses a 5-point Likert-type response scale. The total score ranges from 0 to 100, with higher scores indicating more decisional conflict; a score of 32 and above represents decisional conflict [42]. Role was operationalized as two categories: (1) women (all of those seeking to become pregnant identified as women) or (2) supporting partners (2 identified as women, the rest identified as men).

Clinical characteristics that were abstracted from the electronic health record at six years by a member of the study team (CJ) included gravity, parity, and diagnosis. Both family-building path and parenting outcome were self-reported and confirmed by reviewing the health record. Family-building path was operationalized into three categories: (1) IVF, (2) any other treatments but not IVF including IUI, surgery, and medications, or (3) no treatments. Parenting outcome was operationalized into four categories: (1) added a child through assisted reproductive treatment, (2) added a child through fostering/adoption, (3) added a child naturally without assistance, or (4) did not add a child.

### Quantitative analysis

Descriptive statistics are presented for all variables. We used scatter plots to show the distribution of decision regret, separately by parental role, family-building path pursued, and outcomes. Analysis of Variance (ANOVA) was used to determine if decision regret was significantly different by role, treatment, or outcome, using STATA [43]. Correlations were used to describe the relationship between decisional conflict at baseline and decision regret at six years. Differences between groups were considered statistically significant at *P* < *0.05*.

### Qualitative analysis

We conducted a systematic content analysis of the responses to the open-ended questions on the survey according to standard processes [44]. The team used both inductive and deductive coding [45, 46]. Deductively, common themes were organized by Connolly and Reb’s framework for sources of regret in health care, namely satisfaction or regret related to process and satisfaction or regret related to outcomes [33]. Coders then allowed thematic patterns to emerge inductively from the participants responses. Three members of the research team categorized responses according to the coding scheme using NVivo [47], with 100% of open-ended responses double coded. Team meetings were held to discuss and resolve inconsistencies in coding [30].

## Results

At baseline, 92 women and 68 partners enrolled in the study. At six years, 76 women and 48 partners participated (comprising 45 couples and 34 individuals), representing a 78% retention rate, with similarities by age, race, socioeconomic status, and diagnosis of male-factor infertility between those who did and did not respond at 6 years. However, of those who agreed to be contacted but did not complete the six-year questionnaire (n = 36), 34% were diagnosed with a female factor infertility (vs. 80% of responders), 38% had not pursued any treatment (vs. 20% of responders), and 62% had added a child to their family by 1 year (vs. 88% of responders). Four participants did not complete the decisional regret scale, making the analytic sample n = 120.

Most participants had a college degree. At six years, mean age was 41 (range 25–62). Most (92%) were Non-Hispanic white, reporting very good (43%) or good (30%) health, and reported a household income of $60,000 or more (Table 1). Almost all participants (97%) were in the same relationship they had been prior to their initial consultation, with an average length of 11 years. Mean gravidity was 2.1, and mean parity was 1.4. Infertility diagnoses were anovulation (36%), diminished ovarian reserve (29%), male factor infertility (29%), uterine factor infertility (14%), endometriosis (11%), unexplained (11%), tubal factor (11%) and recurrent pregnancy loss (9%). Two-thirds of participants had added a child to their family using treatment, 16% added a child through fostering/adoption, 7% without any treatment, and 12% did not add a child to their family.

**Table 1:** Sample characteristics (n = 120)

	Total (n = 120)	Women (n = 72)	Partners (n = 48)
*Sociodemographics, % (n)*			
Education at baseline			
High School or less	7 (5.9)	3 (4.2)	4 (8.3)
Some College/Associates degree	21 (17.8)	10 (13.9)	11 (22.9)
College degree	51 (43.2)	31 (44.3)	20 (41.7)
Advanced degree	39 (33.1)	26 (37.1)	13 (27.1)
Age at 6 yr follow up, mean (SD)	40.8 (6.3)	39.6 (5.4)	42.5 (7.0)
Race/Ethnicity at baseline			
Non-Hispanic, white	110 (91.7)	64 (88.9)	46 (95.8)
Hispanic and/or non-white	10 (8.3)	8 (11.1)	2 (4.2)
General Health at baseline			
Excellent	25 (20.8)	19 (26.4)	6 (12.5)
Very good	52 (43.3)	35 (48.6)	17 (35.4)
Good	36 (30.0)	18 (22.2)	20 (41.7)
Fair	7 (5.8)	2 (2.8)	5 (10.4)
Household Income at baseline			
$39,999 or less	5 (4.2)	3 (4.12	2 (4.3)
$40,000–$59,999	8 (6.7)	4 (5.6)	4 (8.5)
$60,000–$79,999	15 (12.6)	11 (15.3)	4 (8.5)
$80,000–$99,999	14 (11.8)	9 (12.5)	5 (10.6)
$100,000–$199,999	62 (52.1)	36 (50.0)	24 (55.3)
$200,000 or more	15 (12.6)	9 (12.5)	6 (12.8)
*Relationship*			
In same relationship as at baseline			
Yes	112 (97.4)	65 (95.6)	48 (100)
No	3 (2.6)	3 (4.4)	0
Length of relationship at 6 year follow up, years	11.4 (3.5)	11.5 (3.6)	11.2 (3.7)
Gravity at 6 year follow up, mean (range)		2.1 (0–7)	
Parity at 6 year follow up, mean (range)		1.4 (0–4)	
Diagnosis*			
Female Factor	95 (79.2)	56 (77.8)	39 (81.3)
Male Factor	31 (25.8)	17 (23.6)	14 (29.2)
Unexplained	16 (13.3)	10 (13.9)	6 (12.5)
Treatments*			
No treatments	20 (16.6)	15 (20.8)	5 (10.9)
Medications only	48 (40.0)	29 (40.3)	19 (39.6)
IUI	61 (50.8)	38 (52.8)	23 (47.9)
IVF	21 (17.5)	11 (15.3)	10 (20.8)
No donor	30 (24.2)	17 (14.2)	13 (10.8)
Donor egg	3 (2.5)	2 (1.7)	1 (.8)
Surgery	29 (24.2)	15 (20.8)	14 (29.2)
Female Pelvic Surgery	32 (26.7)	19 (15.8)	13 (10.8)
Male Surgery	22 (18.3)	9 (7.5)	13 (10.8)
Parenting Intentions			
Currently Trying	21 (17.8)	15 (20.8)	6 (12.5)
Currently Expecting^a^	14 (11.7)	8 (11.1)	6 (12.5)
Parenting Outcomes			
Added a child through treatment	79 (65.8)	46 (63.9)	33 (68.9)
Added a child through adoption or fostering	19 (15.8)	12 (16.7)	7 (14.6)
Added a child without assistance	8 (6.7)	5 (6.9)	3 (6.3)
Did not add a child	14 (11.7)	9 (12.5)	5 (10.4)
Decisional Conflict Scores at Baseline	42.8 (19.4)	41.4 (18.1)	45.2 (21.1)

^*^Percentages for treatments and diagnoses sum to greater than 100% because participants may have undergone more than one type of treatment and/or been diagnosed with male and female factor infertility

^a^Currently pregnant or expecting a child through adoption

### Decision regret by role

Average decision regret was similar by role (Table 2). The ANOVA testing differences in decision regret scores by role was not significant (F = 0.08; p = 0.77). Half of women and half of partners expressed no regret, 30–35% expressed some regret, and 19% of women and 15% of partners expressed moderate-to-severe regret (score > 25, Table 2). Among those expressing moderate-to-severe regret, there were two couples in which both people had high regret scores (one couple had scores of 90 and 55 and a second couple had scores of 80 and 35). One of these couples had one unsuccessful cycle of IUI and one unsuccessful cycle of IVF, with both people later expressing dissatisfaction with their medical care from lack of screening for diabetes prior to treatment. The other couple had six unsuccessful cycles of medications, and the partner later wondered if his wife’s subsequent breast cancer diagnosis was related to fertility treatments, leading to his high regret.

**Table 2:** Decision regret 6 years after initial consultation, by role (n = 120)

Decision Regret Scale Score	Total (n = 120)	Women (n = 72)	Partners (n = 48)	Chi-square or T-test p-value
Mean, (SD)	11.2 (17.3)	10.8 (16.4)	11.8 (18.9)	0.36; p = 0.72
Median	0	0	0	
Range	0–90	0–70	0–90	
No Regret, n (%)	61 (50.8%)	37 (51.4%)	24 (50%)	
Moderate-to-Severe Regret, n (%)	21 (17.5%)	14 (19.4%)	7 (14.6%)	0.27; p = 0.60

Score of 0 indicates no regret; Moderate-to-severe regret or more is indicated by a score of 25 or more

### Decision regret by treatments and outcomes

**Fig. 1:**
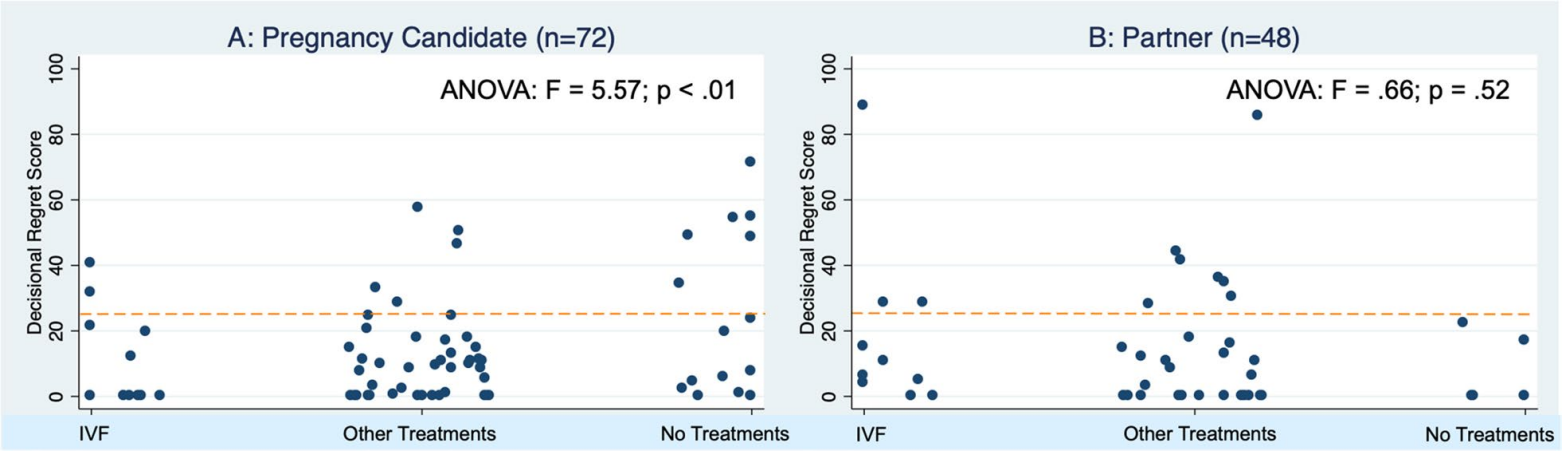
Decisional regret scores by treatment and role

**Fig. 2:**
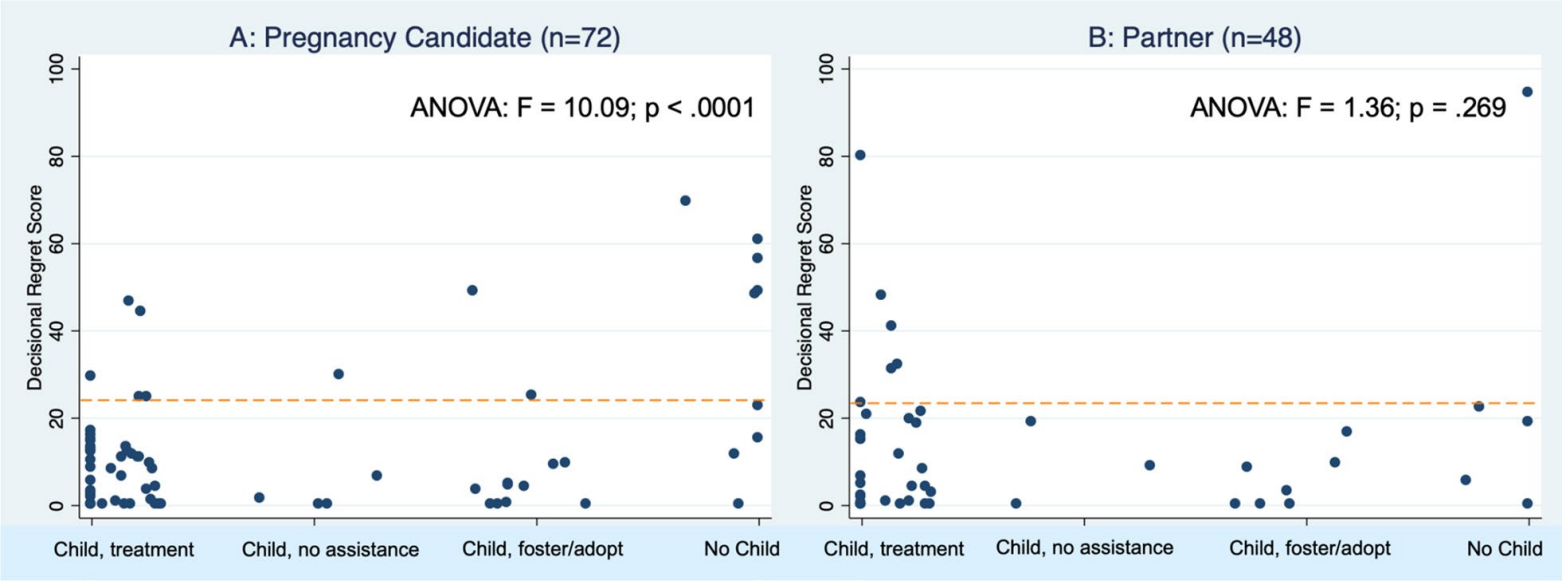
Decisional regret scores by pregnancy outcome and role

When examined by treatment category, for women, there was higher regret among those who did not have treatment (median 15) compared to those who had IVF or other treatments (medians 0, Fig. 1A). Women who did not add a child to their family expressed much higher regret (median 35) compared to those who added a child through foster, adoption, or no medical treatments (median 5) and those who added a child through treatment (median 0) (Fig. 2A). These differences were statistically significant (F = 10.09, p < 0.001) for women but not for men (F = 1.36, p = 0.269).

For partners, when examined by treatment category, the distribution was similar regardless of treatment category, with those expressing moderate-to-severe regret evenly distributed by treatment (Fig. 1A). Likewise, the distribution of regret by outcome was similar regardless of whether they added a child to their family through treatment, through other means, or not at all. A male partner who had tried IVF and had not added a child to his family expressed the highest regret (score of 90).

### Baseline decisional conflict and decision regret

Before the initial specialty consult, women had lower average decisional conflict regarding family-building than partners (41 vs. 45) though this was not statistically significant (p = 0.238). Correlations between decisional conflict before the initial infertility consultation and decision regret six years later was low: the total Pearson correlation was 0.03 (p = 0.715); among women only the correlation was 0.07 and among partners only it was 0.01.

### Reflections on types and cost of treatments

Nearly all participants, 90%, reported being happy with the medical treatments they chose. Eleven participants (9%) wished they had tried more medical treatments, 12 participants (10%) wished they had tried fewer medical treatments, and six participants (5%) wished they had tried different medical treatments (Additional file 1: Table S1). Participants were able to endorse more than one statement, and many endorsed both being happy with the treatments they chose and wishing they had tried more (n = 7, 6%), fewer (n = 6, 5%), or different (n = 2, 2%) treatments. Most participants (80%) were also satisfied with how much money they spent trying to add a child to their family (Additional file 1: Table S1). Seven percent of participants wished they had spent more money, while 28% of participants wished they had spent less. Again, participants were able to endorse more than one statement, and some reported being satisfied with how much money they spent but also wishing they had spent more (n = 6, 6%) or less money (n = 17, 14%). Participants who achieved a live birth through fertility treatments were more likely to be happy with the treatments they chose (99% vs. 80%, p < 0.001) and more likely to have wished they had spent less money on treatments (38% vs. 16%, p = 0.008) compared to those who did not achieve a live birth using fertility treatments. Similarly, participants who were a parent or guardian to a child (including fostering or adoption) were more likely to be happy with the treatments they chose (93% vs. 73%, p = 0.04) and less likely to have wanted to try more treatments (6% vs 27%, p = 0.029) or different treatments (3% vs. 20%, p = 0.024) compared to those who were not a parent or guardian to a child.

### Qualitative themes

Participants described the process of trying to add a child to their family as mentally, emotionally, physically, and financially burdensome, though for those who were able to add a child to their family, many said the effort was worth it (Table 3). Many participants took the opportunity to express gratitude for the ability to add a child to their family, whether through assisted reproductive technology, without assistance, or through fostering/adoption. Others expressed dissatisfaction or regret about the family-building process, with financial concerns, frustration with the medical care received, and health-related concerns (i.e., cancer) of particular importance. Finally, there were some participants who reported being satisfied with their outcomes, even though they did not add a child to their family.

**Table 3:** Qualitative Themes and Example Quotes

Theme	Theme description	Example Quote(s)
Burdensome process of adding a child to family was “worth it”	Participants acknowledge the physical, mental, and emotional difficulties involved in the process of adding a child to one’s family while also expressing appreciation and sentiments that “it was all worth it.”	“It was no question with my husband and I that we wanted to add to our family, it was just a matter of how to, either through treatment or adoption. It was SO (like I can’t even tell you how much) exhausting mentally, emotionally and financially. But after 6 years, 5 cycles of IVF, we have our twin girls and another on the way, and it was worth it. Every single procedure, test, vial of blood, appointment, phone call, every dollar, was worth it.” Woman, age 35 “I feel very grateful that my husband and I were able to adopt our son. We tried 6 rounds of IUI treatments and we were left with great disappointment every time it didn’t work. Adopting was the best decision we could have made; it helped us have our family, helped the birth mother, and it helped our son find his forever home.” Woman, age 38 “It was a hard process in the beginning with our fertility struggles and the emotional roller coaster but I do not regret for one second that we went through that to get our children.” Woman, age 31 “I think it turned out in the end; it was a tough battle but worth it.” Male partner, age 41 “All the effort it took was worth it. Our 2 children are the greatest accomplishment of our lives.” Male partner, age 30 “We are extremely happy to have added our son to our family through adoption. The process itself was horrendous and emotionally exhausting, but it all worked out.” Woman, age 35 “We have two beautiful children and they were worth the process for sure. Adoption would not have been any less expensive.” Woman, age 45
Gratitude for parenthood	Participants express thankfulness for the way they added a child to their family, whether that was through treatment, fostering, adoption, or other paths	“I love the path we've taken to become parents thru fostering. It's extremely fulfilling, and I couldn't imagine not doing something like this.” Male partner, age 40 “We have tried and continue to get ourselves healthy and able to have more children. It has been amazing to know that we have two miracle children, despite our health and fertility struggles.” Male partner, age 31 “Great. We had two kids from the same donor sperm via IUI.” Woman, age 33 “Both adoption and IVF brought children to our family, and so I am extremely grateful for the decisions we made.” Woman, age 33
Dissatisfaction or regret about a past decision or process related to trying to add a child to their family	Participants reflecting on the path they attempted to add a child to their family and expressing discontent in the path	“Should have tried earlier.” Woman, age 45 “I wish I didn’t have to do clomid.” Woman, age 33 and Woman, age 37
Dissatisfaction or regret regarding the expenses to add a child to one’s family	Disappointment or unhappiness expressed related to the financial cost of adding a child to one’s family	“I wish we were able to afford to do more to add children to our family.” Woman, age 40 “I wish I had the financial means to do more to add to our family.” Woman, age 41 “I love my kids, could have done without the financial burden.” Male Partner, 43 “It's absurd how expensive IVF is. Absolutely insane! And I feel at least a portion should be covered by insurance or there should be a specific insurance plan available strictly for infertility. You shouldn't have to go broke to have a family.” Woman, age 40 “I wish we would have done one round of egg retrieval but if it wouldn’t have worked (like the first time) I think we would be worse off financially and emotionally, so I’m ok with it.” Woman, age 40 “We end[ed] up almost spending the same as IVF but [it] took us forever to have a child through inseminations [IUI].” Woman, age 31
Dissatisfaction or regret related to medical care	Disappointment or unhappiness expressed related to participants interaction with the medical system	“Your staff misled us on the viability of our options.” Male Partner, age 55 “I should have trusted my gut that something ELSE was wrong with me. Each time I had a negative side effect (i.e., dizziness, thirst, vision issues, lethargy), I was told by the attending nurse that these were side effects of the treatments I was receiving. I was never allowed to talk to the doctor directly. If only I had reached out to a qualified primary care medical provider, I would have learned of my diabetes earlier and possibly had the opportunity to continue growing my family.” Woman, age 49 “There were times during my treatment when I felt like I was being treated according to a 'one size fits all' plan… Ultimately, I wish I had advocated harder for myself from the get-go. I still would have chosen IUIs, but I would have increased and/or added certain medications to the mix… when fertility treatments are not covered by insurance, there are significant financial repercussions from failed cycles. Messing around with no/low intervention cycles can financially break a patient before they have the opportunity to 'move up' to an intervention that works.” Woman, age 40
Distress or regret because of health	Worry or concern related to participants or participants partner’s health as a result of infertility treatment	“My wife found a lump on her breast when our son was only 4 months old. It turned out to be breast cancer. Knowing what she went through, possibly because of the clomid, if I knew then what I know now, I’d ask her not to take it. But that could mean our son potentially would not have been born. I don’t know.” Man, age 38 “I don’t want to go through the fertility meds and hormones again. I have had multiple friends who have had fertility treatments and later developed breast or ovarian cancer and I am afraid to do anything like this again because of my friends.” Woman, age 33
Distress or regret about not adding a child to their family	Worry or concern related to unsuccessful paths to parenthood	“My husband and I went through a few procedures to conceive. None of the procedures or medication helped. I felt very discouraged and sad that we did not get pregnant.” Woman, age 41
Resignation	Abdication for the outcome of pursuing infertility treatment	“It was just how it had to be.” Woman, age 52 “We tried, it didn't work. Was a little sad, but we have moved on to enjoy life as God has it planned for us.” Woman, age 49
Satisfaction even though they did not add a child to their family	Happiness related to not adding a child to one’s family	“I felt it was a good decision at the time and yes, I [would] totally do it again, I just wish it would have worked. But at this stage in my life now I'm somewhat happy it didn't” Male partner, age 41 “My partner and I broke up, hugely [due] to [trying to have] a baby, and it just made other issues worse that were not even related to that. In a way I'm glad we separated before having a child. I would not want to be a single mother!!” Woman, age 42

## Discussion

This mixed methods longitudinal study on women and their partners who sought reproductive specialty care found that about half expressed regret regarding the decisions they made about how to add a child to their family. To our knowledge, these data present the first mixed methods longitudinal investigation of decision regret among both women and their partners experiencing infertility. There were no significant differences in regret by parental role. Women who did not pursue any treatment had significantly higher regret compared to those who pursued IVF or other treatments. Likewise, women who did not add a child to their family had significantly higher regret compared to women who added a child through treatment, through fostering/adoption, or without any treatment. In contrast, among partners there were no significant differences in regret by treatment or outcome.

Much of the research to date on regret among patients experiencing infertility is specific to the cancer survivorship experience [35, 37, 48] or to those pursuing IVF [49], whereas this study assessed decision regret among those who sought a specialty consult for infertility but who may or may not have pursued any treatment. Though there did not seem to be a pattern among partners between regret and particular paths pursued, there were clear patterns among women seeking to add a child to their family. Women who did not undergo any treatment expressed significantly more regret compared to those who pursued IVF or other fertility treatments. It is possible that those who did not try treatment expressed greater regret because they feel they gave up too easily or did not try hard enough. Indeed, some participants in our study described pursuing treatment because they wanted to prevent future regret.

Our findings were more prominent regarding outcomes: women who did not add a child to their family were much more likely to express regret compared to women who did add a child, whether through treatment, fostering or adopting, or without assistance. This finding is in line with findings from Sundaram and colleagues’ cross-sectional study of 337 people that showed that among patients who underwent IVF, having a live birth was related to significantly lower regret [49]. Importantly, our study extends the finding beyond the context of IVF.

The financial burden of infertility was a common theme, with more than 1 in 4 participants indicating they wished they had spent less money trying to add a child to their family coupled with many spontaneous comments referencing financial concerns. It is important to note that in the state of Wisconsin, there is no insurance fertility mandate and the median household income of the state in 2020 was about $64,000 [50]. Thus, fertility treatments such as IVF or oocyte donation and adoption fees (particularly for international adoption) can pose a significant financial burden for would-be parents. These findings again align with Sundaram et al. work identifying out-of-pocket costs were significantly related to regret among women who underwent IVF [49].

A reoccurring theme at six years was the mental and emotional toll of decision making when trying to add a child to one’s family. Our previous work examining decisional conflict in this sample at 1 year highlighted the mental and emotional burden for those experiencing infertility, suggesting the burden caused a delay in decisions, and decisional conflict took twice as long to resolve among supporting partners [19]. Previous research in other samples has demonstrated a heightened prevalence of anxiety [3, 17, 51] and depression [15, 52] among patients experiencing infertility compared to general populations, with one study showing a relationship between duration of infertility (i.e., time spent trying) and increased anxiety [3]. Importantly, research suggests women experience a larger psychological burden than men, with significantly worse quality of life, depression, and anxiety [4, 53, 54]. These findings may be related to decision fatigue, and suggests the importance of support in the decision-making process.

An important clinical implication from these findings relates to the different results in regret between the women and their partners, highlighting the importance of offering patients and their partners psychosocial support and education that includes multiple paths to parenthood (not just medical treatments) early in the process, as time and finances are limited for most people. Recent research among women in the UK and Portugal showed that the vast majority (90%) were willing to discuss the possibility of IVF treatment being unsuccessful as part of routine care [55]. Among women in our study, any path to parenthood that resulted in adding a child to one’s family was associated with lower regret, while no pattern between path and regret was seen among partners, demonstrating that women and supporting partners experienced regret differently. This is unsurprising given previous findings from this same cohort of patients that showed they entered into the fertility decision making process with different priorities, in particular, more women prioritized becoming a parent and having a child in the next year or two, whereas more men prioritized a genetic connection to their child, maintaining a close relationship with their partner, and avoiding side effects from treatment [18].

Our study had important limitations. It was conducted at a single academic medical center and employed convenience sampling techniques, thus limiting the generalizability of these results. The initial response to the study was modest, due in large part to the nature of recruitment, which had a short turnaround time to complete baseline data collection before a first appointment with a reproductive specialist and did not contact potential participants more than once. There were some differences between participants who did and did not respond to the six year survey, specifically a higher proportion of those who did not respond never pursued treatment and did not add a child to their family. Therefore, these perspectives are likely underrepresented in this analysis. Further, only a small portion of participants remained childless and/or did not pursue treatment, therefore, results on their level of regret or satisfaction may be conservative estimates and larger studies should verify findings. Only a small minority of our participants had insurance coverage for treatment, which may overestimate the relationship between financial impacts and regret. We acknowledge that our study population was relatively homogeneous both racially and socioeconomically. Our sample size was sufficient for our qualitative analyses [56] but limited the statistical power we had to assess quantitative relationships between patient characteristics and regret. Future research would benefit from larger sample sizes to confirm and extend our findings.

Despite these limitations, our study provides a unique investigation into the long term mental and emotional experiences of trying to add a child to one’s family, including potentially regretting certain paths, decisions, or lack of decisions. This study investigates a number of gaps in the literature. First, studies have not broadly investigated decision regret among patients, including men, who sought specialty care for infertility, especially among those who do not ultimately pursue treatment. Second, research has not looked at the long-term prevalence of regret among those experiencing infertility, making our data six years following a consultation with a reproductive specialist novel. Finally, there is a general lack of the male perspective in infertility decision making research. Ultimately, fertility providers' wishes for patients are to help them achieve their family building goals and not regret the decisions they make along the way. Results from this study can provide evidence for fertility providers and counselors to share with patients as they weigh treatment decisions during the arduous course of a fertility journey.

## Conclusions

This longitudinal study provides new insight into the burden of infertility. For women seeking parenthood, any of the multiple paths to parenthood may prevent future decision regret. Understanding the extent to which couples experience regret on their path to parenthood after seeking consultation with a reproductive specialist can improve holistic approaches to patient-centered infertility care. Greater psychosocial, financial, and decision support is needed to help patients and their partners navigate family-building with minimal regret.

### Supplementary Information

**Additional file 1: Table S1.** Reflections on infertility treatments by live birth through ART and any children.

## Data Availability

Data regarding any of the subjects in the study has not been previously published unless specified. Data will be made available to the editors of the journal for review or query upon request.
