# Supplementary material for: Decision regret among couples experiencing infertility: a mixed methods longitudinal cohort study
Source: Reprod Health. 2023 Nov 9;20:165. doi: 10.1186/s12978-023-01699-5 (PMC10633954; doi:10.1186/s12978-023-01699-5)
Supplement: Supplementary file 1 — Additional file 1: Table S1. Reflections on infertility treatments by live birth through ART and any children. [file 12978_2023_1699_MOESM1_ESM.docx]

**Table S1. Reflections on infertility treatments by live birth through ART and any children**

|  | **Responded “Yes”** | | | | | | |
| --- | --- | --- | --- | --- | --- | --- | --- |
|  | Total  (n=124) | Live Birth through ART (n=69) | No Live Birth through ART (n=55) | p-value | Any Children  (n=108) | No Children  (n=15) | p-value |
| I wish I had tried more medical treatments (n=123) | 11 (9%) | 3 (4%) | 8 (15%) | 0.058 | 7 (6%) | 4 (27%) | **0.029** |
| I wish I had tried fewer medical treatments (n=123) | 12 (10%) | 5 (7%) | 7 (13%) | 0.29 | 11 (10%) | 1 (7%) | >0.99 |
| I wish I had tried different medical treatments (n=123) | 6 (5%) | 2 (3%) | 4 (7%) | 0.40 | 3(3%) | 3 (20%) | **0.024** |
| I am happy with the medical treatments that I chose (n=123) | 111 (90%) | 68 (99%) | 43 (80%) | **<0.001** | 100 (93%) | 11 (73%) | **0.040** |
| I wish I had spent more money on trying to add a child to my family (n=124) | 9 (7%) | 2 (3%) | 7 (13%) | 0.076 | 8 (7%) | 1 (6%) | >0.99 |
| I wish I had spent less money on trying to add a child to my family (n=123) | 35 (28%) | 26 (38%) | 9 (16%) | **0.008** | 34 (32%) | 1 (6%) | **0.038** |
| I am satisfied with how much money I spent trying to add a child to my family (n=122) | 98 (80%) | 55 (82%) | 43 (78%) | 0.59 | 87 (82%) | 11 (69%) | 0.31 |
